# Genome Analysis, Metabolic Potential, and Predatory Capabilities of Herpetosiphon llansteffanense sp. nov.

**DOI:** 10.1128/AEM.01040-18

**Published:** 2018-10-30

**Authors:** Paul G. Livingstone, Russell M. Morphew, Alan R. Cookson, David E. Whitworth

**Affiliations:** a Institute of Biological Environmental and Rural Sciences, Aberystwyth University, Ceredigion, United Kingdom; McMaster University

**Keywords:** antimicrobials, biological control, comparative genomics, myxobacteria, predator, prey

## Abstract

Herpetosiphon spp. are ubiquitous, chemoheterotrophic, filamentous gliding bacteria with the ability to prey on other microbes through a “wolf pack” mechanism. The genus currently comprises four known species (H. aurantiacus, H. geysericola, H. giganteus, and H. gulosus), which produce antimicrobial secondary metabolites such as siphonazole. As part of a study isolating myxobacterial wolf pack predators, we serendipitously isolated a novel environmental strain (CA052B) from the edge of a stream at Llansteffan, United Kingdom, which was identified as a member of the Herpetosiphon genus. A lawn culture method was utilized to analyze the predatory activity of CA052B against 10 prey organisms of clinical relevance. CA052B was found to prey on Escherichia coli, Klebsiella pneumoniae, Proteus mirabilis, Staphylococcus aureus, Staphylococcus epidermidis, Staphylococcus saprophyticus, Enterococcus faecalis, Bacillus subtilis, and Candida albicans. Purified CA052B outer membrane vesicles also exhibited killing activity against the prey organisms when tested by flow cytometry. 16S rRNA sequencing of CA052B showed 98 to 99% identity with other Herpetosiphon species members. Comparing the genome of CA052B with the publicly available genomes of H. aurantiacus and H. geysericola revealed average nucleotide identities of only 84% and 91%, respectively, whereas the genome-to-genome distance calculation showed sequence identities of 28.2% and 46.6%, respectively. Biochemical characterization also revealed distinctions between CA052B and both H. gulosus and H. giganteus. Thus, strain CA052B^T^ (= DSM 107618^T^ = NBRC 113495^T^) is proposed to be the type strain of a novel species, Herpetosiphon llansteffanense sp. nov. The genome sequence of CA052B also revealed diverse secondary metabolite biosynthetic clusters, encouraging further exploration of its antibiotic production potential.

**IMPORTANCE** Predatory bacteria are able to kill and consume other microbes and are therefore of interest as potential sources of new antimicrobial substances for applications in the clinic. “Wolf pack” predators kill prey by secreting antimicrobial substances into their surroundings, and those substances can kill prey organisms independently of the predatory cells. The genus Herpetosiphon exhibits wolf pack predation, yet its members are poorly described compared to other wolf pack predators, such as the myxobacteria. By providing a thorough characterization of a novel Herpetosiphon species, including its predatory, biochemical, and genomic features, this study increases our understanding of genomic variation within the Herpetosiphon genus and how that variation affects predatory activity. This will facilitate future rational exploitation of genus members (and other wolf pack predators) as sources of novel antimicrobials.

## Abstract (precis)

Predatory bacteria are able to kill and consume other microbes and are therefore of interest as potential sources of new antimicrobial substances for applications in the clinic. “Wolf pack” predators kill prey by secreting antimicrobial substances into their surroundings, and those substances can kill prey organisms independently of the predatory cells. The genus Herpetosiphon exhibits wolf pack predation, yet its members are poorly described compared to other wolf pack predators, such as the myxobacteria. By providing a thorough characterization of a novel Herpetosiphon species, including its predatory, biochemical, and genomic features, this study increases our understanding of genomic variation within the Herpetosiphon genus and how that variation affects predatory activity. This will facilitate future rational exploitation of genus members (and other wolf pack predators) as sources of novel antimicrobials.

## INTRODUCTION

Herpetosiphon is a genus of chemoheterotrophic filamentous gliding bacteria with the potential to prey on other microbes (1). Genus members are ubiquitous saprophytic bacteria and can be isolated from freshwater, soil, and marine sources (2). The genus belongs to the phylum Chloroflexi, order Herpetosiphonales, and family Herpetosiphonaceae, with four species within the genus having been described previously, namely, H. aurantiacus, H. geysericola, H. giganteus, and H. gulosus (3).

Members of the Herpetosiphonales order are Gram negative with a cell wall made up of peptidoglycan with the *meso*-diaminopimelic acid replaced by l-ornithine (2). They are monoderms, lacking the lipopolysaccharide (LPS) outer membrane, measure up to 500 μm in length, and are enclosed within a sheath (2). Herpetosiphon spp. are aerobic organisms, growing best at 28°C, with swarming colonies that are orange to red due to the production of carotenoid pigments (4). They exhibit catalase and oxidase activities, can hydrolyze gelatin, starch, casein, and tributyrin, but do not degrade cellulose or reduce nitrates (5). The complete genome of the H. aurantiacus type strain 114-95^T^ is 6.79 Mbp, with 5,577 protein-encoding and 77 RNA genes, a G+C content of 50.9%, and two circular plasmids (4).

Being saprophytic organisms, Herpetosiphon spp. prey on a multitude of organisms through a “wolf pack” strategy involving secretion of a variety of hydrolytic enzymes (6). The H. aurantiacus genome sequence revealed two polyketide synthase (PKS), four nonribosomal peptide synthase (NRPS), five hybrid PKS/NRPS, and three bacteriocin biosynthetic gene clusters (4). Although they are known to produce secondary metabolites with an antimicrobial nature, very few studies have looked at Herpetosiphon spp. as producers of natural products (7). Two Herpetosiphon sp. metabolites, siphonazole and auriculamide, have been shown to possess antimicrobial activity (8, 9), while diterpenes homologous to those found in Mycobacterium tuberculosis have also been reported to be produced by H. aurantiacus (10).

While attempting to culture bacterial predators from soil, we have isolated a novel Herpetosiphon strain (CA052B). Here, we report its characterization as a member of a novel species, Herpetosiphon llansteffanense sp. nov., provide a draft genome sequence, and describe its predatory activity against nine clinically important microorganisms.

## RESULTS

### Strain CA052B exhibits defining features of members of the Herpetosiphon genus.

**FIG 1:**
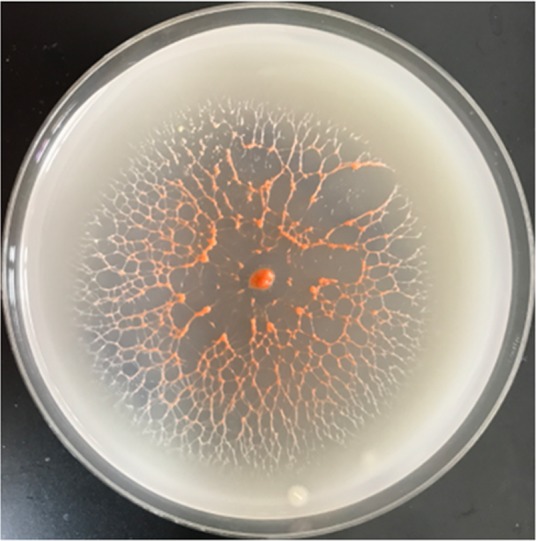
A spreading colony of CA052B on VY-2 agar after 1 week of growth. VY-2 medium contains yeast cells, which are lysed by CA052B as it grows outwards. Note the orange pigmentation of older cells in the center of the plate due to the accumulation of carotenoids.

Strain CA052B was isolated from soil at the edge of a stream at Llansteffan, United Kingdom, using cells of Escherichia coli as the sole nutrient source. Colonies of isolated CA052B appeared moist and became orange pigmented with age. Spreading growth was “membranous,” with aggregation of mounds at the leading edge of the colony becoming connected by veins and filling the entire plate within a week of incubation (Fig. 1). Growth was faster at 30°C than at 37°C or 42°C and required aerobic conditions; no growth was observed anaerobically, with or without 5 to 10% CO_2_. Confluent growth was observed on VY-2 agar, while poor growth could be seen on Mueller-Hinton agar. No growth was observed on blood agar, chocolate agar, or cystine-lactose-electrolyte-deficient (CLED) agar (data not shown). When grown in liquid culture in Casitone yeast extract (CYE), CA052B grew as spherical clumps.

**FIG 2:**
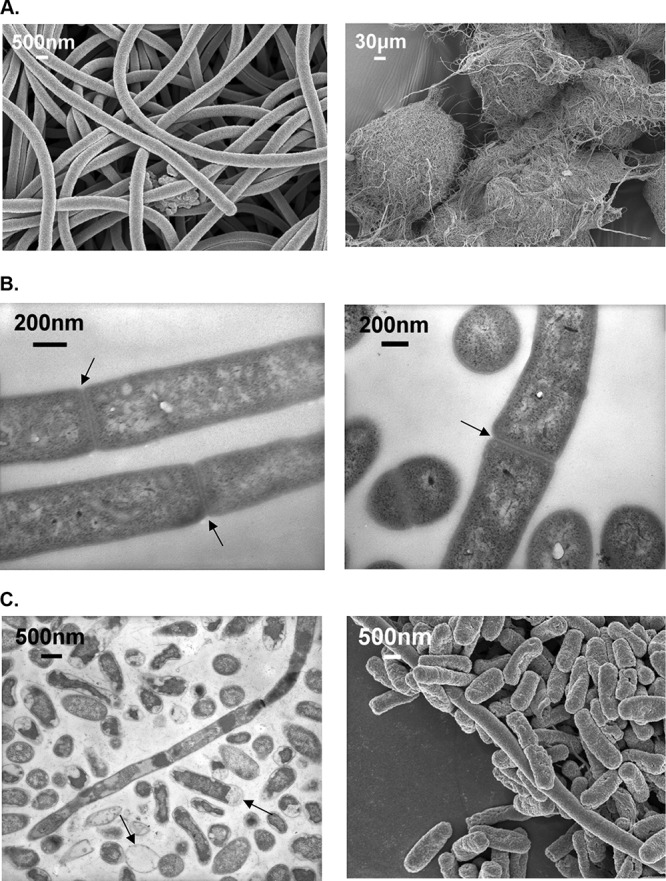
(A) Scanning electron microscope (SEM) images of CA052B cells (left) and aggregates (right). (B) Transmission electron microscope (TEM) images of CA052B. Constrictions between adjoining cells are indicated with arrows. (C) TEM (left) and SEM (right) images of CA052B/Escherichia coli cocultures. Arrows indicate “ghost” prey cells with degraded cytoplasmic contents (left).

Cells of strain CA052B are unbranched filaments without flagella, measuring more than 500 μm in length, that are made up of bacilli in chains, appearing like spaghetti on the scanning electron microscope (SEM) and as tangled aggregates of filaments at larger scales (Fig. 2A). Transmission electron microscopy (TEM) showed that the cells are arranged in long chains with distinct cell walls (Fig. 2B).

### CA052B has a distinctive biochemical activity profile and 16S rRNA sequence.

Biochemical characterization of CA052B was undertaken using the Gram-negative Crystal kit and the API 20E kit (Table 1). CA052B was found to ferment glucose and no other sugars, but it could act on a wide variety of *p*-nitrophenyl sugar derivatives. Table 2 compares the major distinguishing biochemical activities exhibited by CA052B and Herpetosiphon sp. type strains (3). CA052B was more restricted than other Herpetosiphon spp. in its ability to ferment sugars but shared with H. giganteus the ability to metabolize esculin and gelatin.

**TABLE 1:** Results of biochemical character assays for CA052B

Kit	Test	Result
Crystal	Arabinose	−
	Mannose	−
	Sucrose	−
	Melibiose	−
	Rhamnose	−
	Sorbitol	−
	Mannitol	−
	Adonitol	−
	Galactose	−
	Inositol	−
	*p*-Nitrophenyl phosphate	**+**
	*p*-Nitrophenyl α-β-glucoside	**+**
	*p*-Nitrophenyl β-galactoside	**+**
	Proline nitroanilide	−
	*p*-Nitrophenyl bis-phosphate	**+**
	*p*-Nitrophenyl xyloside	**+**
	*p*-Nitrophenyl α-arabinoside	**+**
	*p*-Nitrophenyl phosphorylcholine	−
	*p*-Nitrophenyl β-glucuronide	−
	*p*-Nitrophenyl *N*-acetylglucosaminide	**+**
	γ-l-Glutamyl *p*-nitroanilide	**+**
	Esculin	**+**
	*p*-Nitro-dl-phenylalanine	−
	Urea	−
	Glycine	−
	Citrate	−
	Malonate	−
	Tetrazolium	−
	Arginine dihydrolase	−
	Lysine decarboxylase	−
API 20E	*o*-Nitrophenyl-β-d-galactopyranoside	−
	Arginine dihydrolase	−
	Lysine decarboxylase	−
	Ornithine decarboxylase	−
	Citrate	−
	Hydrogen sulfide	−
	Urease	−
	Tryptophan deaminase	−
	Indole production	−
	Voges-Proskauer	−
	Gelatinase	**+**
	Glucose	**+**
	Mannose	−
	Inositol	−
	Sorbitol	−
	Rhamnose	−
	Sucrose	−
	Melibiose	−
	Amygdalin	−
	Arabinose	−

**TABLE 2:** Abilities of CA052B and other Herpetosiphon spp. to metabolize distinctive biochemical substrates

Biochemical substrate	Metabolization by^*a*^:				
	H. llansteffanense CA052B	H. aurantiacus DSM 785	H. geysericola DSM 7119	H. gulosus DSM 52871	H. giganteus DSM 589
Esculin	+	−	+	+	+
Arginine	−	+	−	+	+
Gelatin	+	−	−	−	+
Glucose	+	+	−	+/−	+/−
Arabinose	−	+	+	+	+
Sucrose	−	+	+	+	+
Rhamnose	−	+	+	+	+
Mannitol	−	+	+	+	+

a Data for strains other than *H*. llansteffanense CA052B are from reference 3.

An internal ∼1,350 bp portion of the CA052B 16S rRNA gene was amplified and sequenced. A search of the CA052B sequence against the EzTaxon database showed a 98.65% similarity hit to H. aurantiacus DSM 785, 99.22% similarity to H. gulosus DSM 52871, 99.11% similarity to H. giganteus DSM 589, and 98.26% similarity to H. geysericola DSM 7119. The 16S rRNA gene sequences from CA052B and all Herpetosiphon sp. type strains were compared pairwise by using EzTaxon (Table 3). In all cases but one, the sequences from Herpetosiphon species strains were more similar to those of Herpetosiphon strains of other species than they were to that of CA052B (H. gulosus was equally similar to both H. giganteus and CA052B), indicating that CA052B belongs to a separate and novel species of Herpetosiphon.

**TABLE 3:**
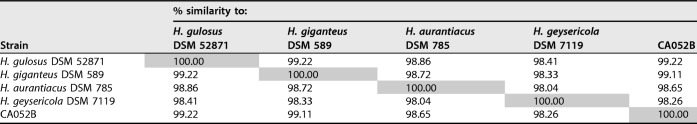
Percent similarities of aligned ∼1,350-bp 16S rRNA gene sequences for CA052B and representatives of the four Herpetosiphon species

### CA052B is an efficient predator of diverse pathogenic prey organisms.

The predatory prey range of CA052B was investigated by testing CA052B for activity against 10 clinically relevant organisms, which included Gram-negative bacteria, Gram-positive bacteria, and a yeast. In a lawn culture assay in which a CA052B inoculum was added onto lawns of the potential prey organism, zones of predation were measured on days 3 and 7 of incubation (Table 4). CA052B preyed particularly well upon E. coli, Klebsiella pneumoniae, Proteus mirabilis, Staphylococcus aureus, Staphylococcus epidermidis, Staphylococcus saprophyticus, and Enterococcus faecalis, with a mean predation zone of 51.4 mm after 7 days. Predation of Bacillus subtilis and Candida albicans was less efficient, with a mean predation zone size of 17.0 mm on day 7. There was no zone of predation seen with Pseudomonas aeruginosa (Table 4). In a previous study, we evaluated the predatory activities of 113 myxobacterial predators against the same panel of prey organisms (11). For three prey organisms (S. aureus, S. epidermidis, and E. faecalis), CA052B produced a larger zone of predation after 3 days than the mean myxobacterium had achieved after 4 days, suggesting particularly efficient predation by CA052B.

**TABLE 4:** Predation assays for 10 potential prey organisms

Prey organism	Zone of predation (mm) generated by:			% of prey cells killed by^*b*^:	
	CA052B		Mx mean, day 4^*a*^		
	Day 3	Day 7		OMVs	SN
Escherichia coli	16	60	16.2	>78	<24
Pseudomonas aeruginosa	No zone	No zone	11.8	ND^*c*^	ND
Proteus mirabilis	11	42	16.6	>75	<10
Klebsiella pneumoniae	11	45	19.7	>82	<20
Staphylococcus aureus	15	43	12.2	>72	<18
Staphylococcus epidermidis	20	59	14.9	>77	<10
Staphylococcus saprophyticus	8	54	11.2	>5	<29
Enterococcus faecalis	20	57	14.2	>51	<16
Bacillus subtilis	11	20	16.2	ND	ND
Candida albicans	9	14	18.2	ND	ND

a Mx mean is the mean predation zone size for 113 myxobacterial isolates after 4 days of incubation, as described previously (11).

b CA052B OMVs and culture supernatant (SN) were tested for predatory activity by flow cytometry.

c ND, not determined.

**FIG 3:**
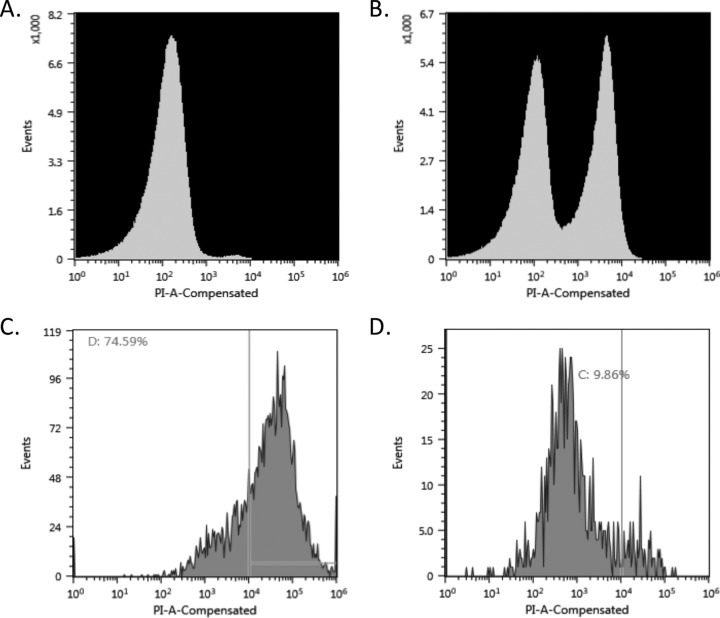
Flow cytometric analysis of prey cell killing by CA052B OMVs and supernatant. (A) Live P. mirabilis cells. (B) P. mirabilis cells treated with ethanol to produce a mixture of live and dead cells. (C) Live P. mirabilis cells treated with CA052B OMVs. (D) Live P. mirabilis cells treated with CA052B supernatant. Percentages of events exceeding the propidium iodide fluorescence intensity (PI-A-Compensated) thresholds, shown as vertical lines, are presented in panels C and D.

The wolf pack predator Myxococcus xanthus secretes outer membrane vesicles (OMVs), and both the OMVs and culture supernatant components purified from M. xanthus cultures have been shown to be capable of killing prey organisms (12, 13). The cytotoxic activities of CA052B OMVs and supernatant were therefore tested for the seven prey organisms against which CA052B showed effective killing in lawn culture assays (Table 4) using live/dead staining coupled with flow cytometry (Fig. 3). Populations of control live and dead prey cells gave distinctive dispersal patterns, and addition of OMVs killed more than 50% of live prey cells (for all seven species). A live/dead gate was defined at the minimum between the live and dead population maxima for each prey (see Fig. 3C for an example), thus underestimating the true size of the dead population (the larger of the two populations) and the percentage of killing (Table 4). Supernatant samples were much less active than OMVs, resulting in prey populations that were dominated by live cells. Gating therefore overestimated the true size of the dead population and the percentage of killing (Table 4). Interestingly, the profile of prey susceptibility to supernatant was different from that to OMVs. For instance, S. saprophyticus was relatively recalcitrant to killing by CA052B cells and its purified OMVs. However, S. saprophyticus was the organism most susceptible to supernatant-mediated killing (Table 4).

### Genome sequence of CA052B.

A draft genome sequence was obtained for CA052B. The draft genome comprised 6,140,944 bp of sequence with a 50.8% GC content spread over 170 contigs, with an N50 of 107,039 bp and an L50 of 20 (i.e., the longest 20 contigs together constituted half of the total sequence length, with the 20th-largest contig having a size of 107,039 bp). The RAST-generated annotation of the predicted CA052B proteins is provided in File S1 in the supplemental material. Table 5 gives a comparison of the two publicly available genomes of H. aurantiacus and H. geysericola with that of CA052B. Automated annotation by RAST identified 5,248 protein-coding sequences and 59 RNA genes in the CA052B genome (File S1). Of the 5,248 predicted proteins, 3,081 (58.7%) were assigned to putative functions by RAST, while 2,167 (41.3%) were hypothetical. The genome appears to contain an incomplete prophage of 16.2 kbp on the 9th-largest contig, with a G+C content of 51.81% and 12 protein-coding sequences, of which 8 have annotated functions (rows 1479 to 1491 inclusive [highlighted] in File S1). The antiSMASH tool (14) identified 10 secondary metabolite biosynthetic gene clusters in the CA052B genome, which were predicted to direct synthesis of one PKS, two NRPSs, two hybrid PKS/NRPSs, two bacteriocins, two terpenes, and one thiopeptide (Table 5).

**TABLE 5:** Genome characteristics of the three sequenced Herpetosiphon strains, including the classes of metabolites predicted to be made by each organism^*a*^

Characteristic	H. llansteffanense CA052B	H. aurantiacus DSM 785	H. geysericola DSM 7119
Size (Mbp)	6.14	6.79	6.24
No. of contigs	170	1 (+2 plasmids)	46
% GC content	50.8	50.9	50.7
No. of coding sequences			
Total	5,248	5,577	4,688
t1PKS-NRPS	2	3	2
Thiopeptide	1	1	1
Terpene	2	2	2
Bacteriocin	2	1	2
NRPS	2	4	2
t3PKS	1	1	1
t1PKS		1	
Lantipeptide-t1PKS-NRPS		1	

a Data for H. aurantiacus DSM 785 and H. geysericola DSM 7119 are from references 4 and 54, respectively. t1 and t3 refer to type 1 and type 3, respectively.

The draft H. llansteffanense genome also revealed several genes involved in carotenoid production, including genes encoding phytoene synthase, neurosporene desaturase, phytoene dehydrogenases, and lycopene cyclase, consistent with the observed production of colored carotenoids. No *N*-acyl homoserine lactone (AHL) synthetic genes were identified. However, an AHL hydrolase gene was present, implying the ability to quench AHL-mediated quorum signaling. Signaling protein genes were abundant in the CA052B genome, with 175 two-component system (TCS) genes predicted using the domain-based classification system (15), including genes for 22 hybrid kinases, 41 single-domain response regulators, and one NtrC family, 19 OmpR family, and 11 NarL family response regulators, which are numbers similar to those for proteins encoded in the H. aurantiacus DSM 785 genome. The 93rd-largest contig contained the genes encoding an entire chemosensory system, including homologs of CheA, CheY, CheW, CheB, CheR, and a methyl-accepting chemotaxis protein. The genome also possessed 33 clustered regularly interspaced short palindromic repeat (CRISPR)-associated genes.

Protein-coding genes from all three available Herpetosiphon genomes (CA052B, H. aurantiacus DSM 785, and H. geysericola DSM 7119) were assigned to orthologous groups (orthogroups) using OrthoFinder (16). A total of 14,326 gene products (90.8% of the total) were assigned to 4,548 orthogroups, of which 3,894 included representatives from all three genomes and 3,558 were single-copy orthogroups. Of the 5,299 coding sequences identified by Prokka/OrthoFinder in the CA052B genome, 4,819 were assigned to 4,768 (90.9%) orthogroups and 480 (9.1%, mainly encoding hypothetical proteins) were unassigned. CA052B shared 4,205 orthologs with H. geysericola and 4,079 orthologs with H. aurantiacus, suggesting that CA052B shares a more recent common ancestor with H. geysericola than with H. aurantiacus.

### Genome index-based taxonomic assignment of CA052B.

Using a percentage of 16S rRNA sequence identity (usually 99%) to demarcate species boundaries can be problematic, for instance, with many examples of separate species exhibiting >99% 16S rRNA sequence identity (17), so the genome sequence of CA052B was used to investigate its relationship with those of other Herpetosiphon species. Recent studies have benchmarked an overall genome-related index (OGRI), which uses the average nucleotide identity (ANI) and digital DNA-DNA hybridization (DDH) as minimal criteria for the identification of novel species (18–20). Only two Herpetosiphon genomes (H. aurantiacus DSM 785 and H. geysericola DSM 7119) were publicly available for calculation of ANI and digital DDH values at the time of this study.

The ANI between CA052B and H. aurantiacus was 84%, and that between CA052B and H. geysericola was 91%, substantially less than the 95 to 96% threshold for species demarcation proposed previously (19). Similarly, the DDH value was estimated to be 28.1% between CA052B and H. aurantiacus and 42.8% between CA052B and H. geysericola, below the 70% cutoff suggested for separate species identification. The DDH value between H. geysericola and H. aurantiacus was also 28.1%. Thus, the OGRI analysis supports the proposal that CA052B is a separate species from H. geysericola and H. aurantiacus.

## DISCUSSION

### Herpetosiphon spp.

The genus Herpetosiphon was first described in 1968, and since then, several studies have reported the isolation of Herpetosiphon from various habitats, including soil, freshwater, and marine sources (5, 21). H. aurantiacus was the first described species (5), followed by H. geysericola (21), and recently the latest two species, H. gulosus and H. giganteus, were described (3). Several organisms originally identified as Herpetosiphon species have since been reassigned to different genera based on their phenotypic and phylogenetic properties (22, 23).

Strain CA052B was isolated from a sample taken at the edge of a stream close to where it joined the estuary of the river Tywi in West Wales and was retrieved by an E. coli “baiting” method used routinely to isolate bacteriolytic myxobacteria (11). CA052B did not grow on filter paper plates which are used to isolate cellulolytic myxobacteria, suggesting that they are not cellulose degrading, in accordance with previous studies (5). The colony and cellular morphologies of CA052B are consistent with those of other Herpetosiphon spp. (Fig. 1 and 2), as is the broad range of prey against which predatory activity was observed (Table 4) (1). The presence of a sheath external to the cell wall has been described by several studies of Herpetosiphon spp. but has also been debated based on electron microscopy data (22). The presence of constrictions at the junctions between cells in our images (Fig. 2B) implies the absence of a sheath; however, further devoted microscopy and membrane composition studies would be needed to categorically test for sheath absence.

### Herpetosiphon llansteffanense sp. nov.

Querying the EzTaxon database with the partial 16S rRNA gene sequence of CA052B revealed its closest match to be H. gulosus, with 99.22% sequence identity (Table 3), and phylogenetic trees based on partial 16S rRNA sequence data also showed CA052B grouping with H. gulosus (data not shown). However, the 16S rRNA gene from H. gulosus also showed 99.22% identity to that of H. giganteus, which has been classified as belonging to a different species. Although 16S RNA sequencing is still a widely accepted method for bacterial taxonomic assignment, its use as a gold standard is debatable (17). Since 16S rRNA sequence identity was at the borderline for species identification, it was necessary to draw on other evidence to assess the taxonomic status of CA052B (18–20).

Genome sequence-based analysis is increasingly considered an appropriate standard for taxonomy (24). Calculation of the ANI and *in silico* DDH value using the genome-to-genome distance calculator (GGDC) (25) showed that CA052B is highly divergent from the two published genomes of Herpetosiphon, justifying assigning CA052B to a novel species. Unfortunately, at the time of this study, whole-genome sequences of the two most recently described Herpetosiphon species, H. giganteus, and H. gulosus, were not available for comparison. However, both H. giganteus and H. gulosus were proficiently saccharolytic, utilizing a number of sugars as the sole carbon source, including rhamnose, arabinose, sucrose, galactose, and mannitol (3), while CA052B can grow only on glucose. Consequently, on the basis of genomic differences and distinct biochemical activities, we propose that CA052B belongs to a novel species, designated Herpetosiphon llansteffanense sp. nov. after the sampling location from which it was isolated.

### H. llansteffanense predatory activity.

Isolating bioactive natural products from organisms is difficult because of the resource-intensive methods required and the unstable nature of the natural products themselves. This has driven researchers to investigate alternative strategies for utilizing predatory organisms directly as therapeutics (see, for example, reference 26). Studying the predatory nature of such organisms is therefore required in order to rationally develop such applications. Predation has been widely studied in many organisms, and different predators have been shown to exhibit various predatory strategies (27). The mode of predation exhibited by Herpetosiphon spp. has been described as a “wolf pack” strategy, similar to that of myxobacteria (1, 6), which is characterized by the cooperative secretion of hydrolases and secondary metabolites.

CA052B exhibited effective predation against both Gram-negative and Gram-positive bacteria (particularly against S. aureus, S. epidermidis, and E. faecalis), with C. albicans, P. aeruginosa, and B. subtilis being relatively recalcitrant to predation (Table 4). Transmission electron microscope (TEM) studies of CA052B and E. coli cocultures revealed that prey cells had sustained significant damage (Fig. 2C), with distorted cell walls and loss of cytoplasmic contents. Compromised cell wall integrity would account for such loss of cytoplasmic contents and is consistent with the secretion of hydrolytic enzymes by the predator and its “wolf pack” mode of predation.

### Wolf pack predation via OMVs.

OMVs are spherical membrane-bound structures secreted by bacterial cells and packed with a cargo of hydrolytic enzymes and metabolites. The OMVs and culture supernatant secreted by the wolf pack predator M. xanthus DK1622 have been shown to kill *E. coli and P. aeruginosa* (13, 28). OMVs and supernatant from CA052B cultures were therefore isolated and tested for killing activity against a range of prey microbes. Flow cytometry revealed that OMVs had a profile of killing activity similar to that of CA052B cells, suggesting that isolated OMVs could potentially be used as an antibiotic therapy. OMVs are being developed for use in vaccines against organisms such as Neisseria meningitidis, Francisella tularensis, Burkholderia pseudomallei, and Mannheimia haemolytica, and they are also studied as a potential vehicle for targeted drug delivery against cancer cells (29). However, the application of OMVs from predatory bacteria as potential antibiotics against human pathogens still remains in its infancy (30).

### CA052B shares common features with other wolf pack predators.

There are striking biological similarities between the wolf pack predators H. llansteffanense CA052B and M. xanthus, and not just in their mode of predation. For instance, they both swarm via gliding motility (with genome-encoded chemosensory regulatory systems), and both produce colored carotenoids, particularly in older cells. In myxobacteria, carotenogenesis is a light-induced light-protective phenomenon, which is thought to have evolved to allow slow-moving myxobacteria to survive hunting on surfaces during the day, and perhaps it is for that reason that nonphotosynthetic H. llansteffanense has retained carotenoid synthesis genes from its photosynthetic Chloroflexia ancestors. Perhaps the wolf pack mode of predation requires long-term investment in an area through the secretion of lytic factors, requiring photoprotection during daylight hours. In M. xanthus, carotenoid biosynthesis genes are expressed under the control of a sigma factor/antisigma factor system (CarQ/CarR), a vitamin B_12_-dependent repressor (CarH), and a MerR family repressor/antirepressor system (CarA/CarS) (31, 32). In the CA052B genome, one of the phytoene dehydrogenase genes is immediately adjacent to a gene encoding a sigma factor, while the phytoene synthase gene is located next to a gene for a MerR family regulator (CarH), suggesting potential commonality in the control of transcriptional regulation. However, carotenogenesis in CA052B was constitutive, with no evidence of light induction of pigment production.

CA052B has an exceptionally large complement of TCS signaling pathway genes for a genome of its size (28.5 TCS genes per Mbp). However, this would be typical for a member of the myxobacteria (33). The disproportionate number of TCS genes in myxobacteria has been considered to be a requirement for the complex process of aggregation, fruiting body formation, and communal sporulation. Both organisms aggregate upon nutrient limitation, a phenomenon preceding communal sporulation in M. xanthus (34), although there are no reports of Herpetosiphon spp. sporulating. Several CA052B gene products have annotated functions related to sporulation, including Spo0J and Soj homologs, three SpoIIE family phosphatases, a Spo0M regulator, and a spore peptidoglycan hydrolase. Nevertheless, CA052B aggregates did not produce viable colonies after incubation at 50°C for 1 h, suggesting that they do not produce heat-resistant spores. Both CA052B and M. xanthus have genomic signatures suggesting that they may be sensitive to prey signaling. M. xanthus is able to sense but not produce AHLs, which probably allows it to detect the presence of prey (35), while CA052B has an AHL hydrolase but no AHL synthetic genes, suggesting that it may interfere with prey quorum signaling. Both organisms have several LuxR family response regulators (11 in CA052B and 5 in M. xanthus), which are involved in AHL binding and transcriptional regulation in quorum-signaling organisms (36).

### Genomic basis of predation.

Genome sequences in isolation do not directly add to the understanding of an organism's biology without comparison to other, better understood, proteins and organisms (37). However, when coupled with phenotypic data, commonalities between the genomes of organisms which share similar behavioral traits allow the exploration of genomic explanations for those traits.

Comparative genomics studies have defined a set of genes associated with predatory activity in bacteria (38). Of those predatory genes, orthogroups containing mevalonate kinase, acetyl coenzyme A (acetyl-CoA) acetyltransferase, hydroxymethylglutaryl-CoA lyase, von Willebrand factor, serine protease, tryptophan 2,3-dioxygenase, and NADPH-dependent flavin mononucleotide (FMN) reductase gene products were found to be present in the CA052B, H. aurantiacus, and H. geysericola genomes.

Herpetosiphon species members are known to produce secondary metabolites, and some have been shown to possess antimicrobial activity (7, 9, 39, 40), while genome sequencing has revealed diverse and idiosyncratic sets of biosynthetic gene clusters (40). Predicted secondary metabolite gene clusters from Herpetosiphon were compared with those from myxobacterial genomes. Herpetosiphon genomes encode 10 to 14 clusters producing 6 to 8 classes of compounds, while myxobacterial genomes have 23 to 39 clusters producing 8 to 20 compound classes (see File S2 in the supplemental material). As expected, a phylogenetic tree based on genomic average nucleotide identity (ANI) grouped Herpetosiphon spp. separately from the myxobacteria, as did a tree based on the ANI values of just the predicted biosynthetic gene cluster sequences (File S2). Therefore, the predicted biosynthetic gene clusters (both the complements of different types of clusters and their sequences) from sequenced Herpetosiphon spp. are clearly distinct from those of sequenced myxobacteria.

In addition, secreted hydrolases are thought to be important during wolf pack predation, and while all three genomes possess large numbers of hydrolase genes, there was considerable variability and individuality in the genomic complements between H. aurantiacus, H. geysericola, and H. llansteffanense. This provides a potential explanation for the molecular basis of the broad but patchy prey range observed both in Herpetosiphon spp. and also in the myxobacteria. It would be informative to investigate whether H. llansteffanense employs a “spider web” predatory strategy similar to that of M. xanthus, with constitutive secretion of antimicrobial factors, and whether it induces the same metabolic response to nutrient availability from lysed prey (41).

### Summary.

Herpetosiphon llansteffanense sp. nov. CA052B exhibits effective predatory activity against a broad range of prey microbes, potentially mediated by secreted OMVs. Its genome sequence provides evidence of secondary metabolite production and will be an invaluable aid for investigating the molecular basis of the organism's antimicrobial activity.

### Taxonomy.

Herpetosiphon llansteffanense (llan.stef.an.en′se. N.L. neut. adj. llansteffanense from Llansteffan, reflecting the fact that the new species was isolated near the village of Llansteffan in Wales [51.77°N 4.39°W]).

Colonies are flat, spreading, and rough, with aggregation into raised rhizoid veins on VY-2 agar (0.5% dried baker's yeast, 0.1% CaCl_2_ · 2H_2_O, 1.5% agar). Orange pigments produced. Nonflagellate cells (1.0 to 1.5 by 2.0 to 2.5 μm) form unbranched septated filaments within sheaths, staining Gram negative. Obligate aerobic growth optimal at 30°C. Ferments glucose and esculin but not arginine, mannose, mannitol, inositol, sorbitol, adonitol, rhamnose, sucrose, melibiose, or arabinose. Gelatin liquefaction positive. Cells prey upon Escherichia coli, Klebsiella pneumoniae, Proteus mirabilis, Staphylococcus aureus, Staphylococcus epidermidis, Staphylococcus saprophyticus, Enterococcus faecalis, Bacillus subtilis, and Candida albicans but not Pseudomonas aeruginosa ATCC 27853. DNA G+C content is 50.8 mol%. The draft genome sequence of the organism is available from GenBank (BioProject record number PRJNA434832). Phylogenetically most similar to Herpetosiphon gulosus.

The type strain (CA052B^T^ = DSM 107618^T^ = NBRC 113495^T^) was isolated from soil collected from the edge of a stream near the village of Llansteffan, United Kingdom [gridref 51.77°N 4.39°W].

## MATERIALS AND METHODS

### Isolation and identification.

Strain CA052B was isolated from soil at the edge of a stream at Llansteffan near Carmarthen, West Wales, United Kingdom. The strain was isolated on WCX agar with E. coli bait, which comprised WAT agar (0.1% CaCl_2_ · 2H_2_O, 1.5% agar, 20 mM HEPES) supplemented with 2.5% cycloheximide (WCX) spotted with an E. coli suspension. Small portions of soil samples were placed adjacent to the E. coli spot, and after incubation, swarming predatory colonies consuming the E. coli bait were isolated. Isolate CA052B was identified by 16S rRNA sequencing using primers F27 and R1389 as described previously (11). The 16S rRNA sequence was compared with the EzTaxon database (42) to identity the most similar sequences, while phylogenetic trees were constructed using MEGA 7.0 (43).

### Genome sequencing and analysis.

Whole-genome sequencing of strain CA052B was performed by MicrobesNG (University of Birmingham, UK) on the Illumina HiSeq 2500 platform using 2 × 250 bp paired-end reads. As part of the pipeline at MicrobesNG, the reads were used to identify the closest reference genome using Kraken (44), and a *de novo* assembly was generated using Spades 3.7 (45). The resulting contigs were then annotated using Prokka 1.11 (46) and RAST 2.0 (47). The genomic average nucleotide identity (ANI) (24) was calculated and *in silico* DNA-DNA hybridization analyzed using the genome-to-genome distance calculator GGDC 2.1 (25).

Biosynthetic gene clusters were analyzed using antiSMASH (14), and two-component system (TCS) signaling protein genes were identified using P2RP (48). Clusters of orthologous genes (COGs) were retrieved from the CA052B, H. aurantiacus DSM785, and H. geysericola DSM7119 genomes using the OrthoFinder 1.1.8 stand-alone tool (16). Thirty-one housekeeping genes were acquired using AMPHORANet (49); they were then concatenated and aligned, and a maximum-likelihood tree was plotted using a Jones-Taylor-Thornton model on MEGA 7.0 (43). Prophage sequences were identified using the web tool PHAST (50), and annotation of the prophage sequences was achieved using RAST.

### Growth conditions and biochemical characterization.

CA052B was grown aerobically on VY-2 agar (0.5% dried baker's yeast, 0.1% CaCl_2_ · 2H_2_O, 1.5% agar) and in modified Casitone yeast extract (CYE) broth (1% Casitone, 0.4% yeast extract, 10 mM Tris [pH 8.0], 8 mM MgSO_4_) at 30°C in a shaken incubator (180 rpm). Growth was tested aerobically at 37°C and 42°C, at 37°C in the presence of CO_2_, and anaerobically. Growth was also tested on different media, including blood agar, chocolate agar, cystine-lactose-electrolyte-deficient (CLED) agar, and Mueller-Hinton agar (all from Oxoid Limited, UK). Biochemical characterization was undertaken using the Gram-negative Crystal kit (BD BBL Crystal enteric/nonfermenter ID kit) and the API 20E kit (bioMérieux). Pigment production in the presence or absence (plates covered with aluminum foil) of light was tested on VY-2 plates incubated at 30°C for 4 days. Sporulation was tested by heating cell suspensions at 50°C, 70°C, or 90°C for 1 h and spreading onto fresh VY-2 plates.

### OMVs.

CA052B was grown in CYE broth in baffled flasks at 30°C for 7 days in a shaking incubator (180 rpm) to obtain a smoothly suspended late-exponential-growth-phase culture. The culture was centrifuged for 30 min at 10,400 × *g*, and then the supernatant was transferred to fresh tubes (repeated twice) to remove cells. The supernatant was then subjected to ultracentrifugation at 100,000 × *g* for 80 min. The concentrated outer membrane vesicle (OMV) pellet was resuspended in TM buffer (50 mM Tris [pH 7.8], 10 mM MgSO_4_) and stored at −80°C. Culture supernatant devoid of OMVs was also stored at −80°C. Protein estimation was carried out using Qubit protein assay kits (Life Technologies).

### SEM.

Cell morphology was studied by scanning electron microscopy (SEM). Briefly, the organisms were grown in CYE broth for 3 days, centrifuged at 10,400 × *g* for 15 min, and washed twice with TM buffer. The pellet was fixed with 2.5% glutaraldehyde in 0.1 M sodium cacodylate (pH 7.2) and then with 1% osmium tetroxide in 0.1 M sodium cacodylate (pH 7.2). The sample was then dehydrated in an aqueous alcohol series (30%, 50%, 70%, 95%, and 100%) and critical-point dried with hexamethyldisilazane (TAAB Laboratories Equipment Ltd., Aldermaston, UK). The sample was coated in an agar high-resolution sputter coater using a platinum/palladium target to give a coating layer of 6.0 nm and imaged using a Hitachi S-4700 FESEM microscope.

### TEM.

Transmission electron microscopy (TEM) was used to visualize the morphology of bacterial cells and outer membrane vesicles. Structural changes of CA052B and E. coli cocultures were also imaged. To image whole cells and coculture samples, samples were fixed with a primary fixative of 2.5% glutaraldehyde in 0.1 M sodium cacodylate (pH 7.2), followed by a secondary fixative of 1% osmium tetroxide in 0.1 M sodium cacodylate (pH 7.2). Samples were resuspended in 100 μl 2% agarose solution and left at 4°C overnight before sectioning. Ultrathin 60- to 80-nm sections were cut and double stained with uranyl acetate (Agar Scientific) and Reynolds' lead citrate (TAAB Laboratories Equipment Ltd., Aldermaston, UK) (51, 52). Sections were observed using a JEOL JEM1010 transmission electron microscope (JEOL Ltd., Tokyo, Japan), and the images were photographed using Carestream 4489 electron microscope film.

### Predation assays.

Predation assays were carried out using a lawn culture method (11, 53). Briefly, prey organisms (E. coli ATCC 25922, Klebsiella pneumoniae ATCC 700603, Proteus mirabilis NCTC 10975, Pseudomonas aeruginosa ATCC 27853, Staphylococcus aureus ATCC 29213, Staphylococcus epidermidis NCTC 11047, Staphylococcus saprophyticus [wild strain], Enterococcus faecalis ATCC 29212, Bacillus subtilis ATCC 6633, and Candida albicans NCTC 32) were grown in Luria-Bertani (LB) broth for 16 to 18 h and centrifuged at 10,400 × *g* for 15 min. Cells were then washed with TM buffer (50 mM Tris [pH 7.8], 10 mM MgSO_4_) and a lawn made by spreading onto WAT agar (14-cm plates) and air drying. CA052B was grown in CYE broth at 30°C for 7 days in baffled flasks in a shaking incubator to obtain a dense, smooth cell suspension. Cultures were then centrifuged at 10,400 × *g* for 15 min, the pellet was washed in TM buffer, and 10 μl of resuspended cells was inoculated onto the prey lawn. Plates were incubated for 7 days, and the diameter of the zone of swarming (predation) was recorded on days 3 and 7.

### Flow cytometric assays.

Flow cytometry was performed on a Sony SH800 flow cytometer/cell sorter equipped with four lasers analyzing at a rate of 10,000 events per second, sorting cells at a purity of more than 98%. The cell viability of prey organisms was undertaken using propidium iodide (PI) stain, which stains only nonviable cells. For live and dead prey cell controls, bacterial suspensions (10^8^ cells/ml) of exponential-growth-phase cultures were centrifuged at 10,000 × *g* for 2 min and washed with phosphate-buffered saline (PBS) at pH 7.4. Pellets were resuspended in either 1 ml of PBS (live control) or 1 ml of 70% isopropanol (dead control) for 1 h at 30°C before being centrifuged again at 10,000 × *g* for 2 min and resuspended in PBS. To assay the killing activities of the CA052B OMVs and culture supernatant, 10 μl of the prey bacterial suspensions (at 10^8^ cells/ml) was added to 100 μl of OMVs or culture supernatant and incubated for 1 h at 30°C, and then 1 μl of 20 mM PI was added to each sample (OMV- and supernatant-treated cells and live and dead control cells) and left for 15 min to stain the dead cells. Samples were analyzed on the flow cytometer using the 488-nm argon ion laser and with PI fluorescence collected through a 630-nm filter. The raw data (FCS files) were analyzed using the flow cytometer's built-in software.

### Accession number(s).

The genome sequence of Herpetosiphon llansteffanense CA052B has been deposited at DDBJ/ENA/GenBank (BioSample record number SAMN08578589) under accession number PUBZ00000000 (version PUBZ01000000). H. llansteffanense CA052B has been deposited in the DSMZ and ATCC culture collections under accession numbers DSM 107618 and NBRC 113495, respectively.

## Supplementary Material

Supplemental file 1

Supplemental file 2
